# Characterization of the Immunologic Phenotype of Dendritic Cells Infected With Herpes Simplex Virus 1

**DOI:** 10.3389/fimmu.2022.931740

**Published:** 2022-07-05

**Authors:** Jingjing Zhang, Xingli Xu, Suqin Duan, Yang Gao, Danjing Ma, Rong Yue, Fengyuan Zeng, Xueqi Li, Ziyan Meng, Xinghang Li, Zhenye Niu, Guorun Jiang, Li Yu, Yun Liao, Dandan Li, Lichun Wang, Heng Zhao, Ying Zhang, Qihan Li

**Affiliations:** ^1^ Institute of Medical Biology, Chinese Academy of Medical Sciences & Peking Union Medical College, Kunming, China; ^2^ Yunnan Key Laboratory of Vaccine Research and Development on Severe Infectious Diseases, Kunming, China

**Keywords:** herpes simplex virus type 1, dendritic cells, wild-type strain, attenuated strain M6, HVEM

## Abstract

Due to viral envelope glycoprotein D binding to cellular membrane HVEM receptor, HSV-1 can infect certain dendritic cells, which becomes an event in the viral strategy to interfere with the host’s immune system. We previously generated the HSV-1 mutant strain M6, which produced an attenuated phenotype in mice and rhesus monkeys. The attenuated M6 strain was used to investigate how HSV-1 infection of dendritic cells interferes with both innate and adaptive immunity. Our study showed that dendritic cells membrane HVEM receptors could mediate infection of the wild-type strain and attenuated M6 strain and that dendritic cells infected by both viruses in local tissues of animals exhibited changes in transcriptional profiles associated with innate immune and inflammatory responses. The infection of pDCs and cDCs by the two strains promoted cell differentiation to the CD103^+^ phenotype, but varied transcriptional profiles were observed, implying a strategy that the HSV-1 wild-type strain interferes with antiviral immunity, probably due to viral modification of the immunological phenotype of dendritic cells during processing and presentation of antigen to T cells, leading to a series of deviations in immune responses, ultimately generating the deficient immune phenotype observed in infected individuals in the clinical.

## Introduction

Herpes simplex virus 1 (HSV-1), a member of the alpha group in the herpesvirus family and the agent of oral and genital herpetic diseases (1, 2), has long been a public health concern due to its significance for clinical treatment and pandemic control (3, 4). The disease caused by this agent and its clinical outcomes has substantial effects on the quality of human life (5, 6) due to the complicated mode of viral infection in the human body, as various virus-encoded proteins enable interaction with the immune system during infection (7–9). Previous data suggested that viral proteins could have different interference effects targeting various cells involved in innate and adaptive immunity, including dendritic cells and lymphocytes, and shape the immune response and pathological outcomes (10–13). Dendritic cells, as important innate immune cells, function not only in phagocytosis and uptake of antigens from infected epithelial tissues and presentation to T cells (14, 15) but also in regulation of the relationship between innate immunity and inflammatory reactions for further activation of adaptive immunity (16, 17). Reported data indicated that HSV is capable of infecting some dendritic cells and replicating in those cells. This event is due to the viral envelope glycoprotein D binding to the HVEM receptor in the membrane dendritic cells (18, 19). Based on these findings, a study tracing HSV-1 infection discovered that not only was viral antigen in tissues phagocytized by activated dendritic cells with infected epithelial cells and transferred to lymph nodes for antigen presentation to T cells, but that infected dendritic cells also carried the complete virion to T cells for antigen transfer during the infection process (20, 21), which raised the logical question of how and to what extent dendritic cells infected with HSV-1 could influence the differentiation process and the activationof T cells through specific antigen presentation. Our previous work showed that HSV-1 infection in HVEM^-/-^ mice elicited a disparate immune response phenotype in comparison with that in wild-type mice (unpublished data) and suggested that viral infection of dendritic cells could interfere with innate immune cells, activating adaptive immunity, although the role of dendritic cells in this process is unknown. In the current work, we used the attenuated HSV-1 strain M6, with gene modifications, as a control (22), investigated the possible mechanism of the altered immunological phenotype induced by infected dendritic cells in mice, and explored the relationship of this alteration with the development of specific antiviral immunity during HSV-1 infection. All of the results suggested that viral infection of dendritic cells is an important factor that interferes with the immune response.

## Material and Methods

### Animal and Ethics

Four-to six-week-old female Balb/c mice were purchased from Vital River (Beijing, China) and housed in a specific pathogen-free facility at the Institute of Medical Biology. The room temperature was maintained at approximately 25°C during the experiments. Food and water were readily available. All animals were fully under the care of veterinarians at the Institute of Medical Biology (IMB), Chinese Academy of Medicine Science (CAMS). All animal experiments were performed according to the National Institutes of Health Guide for the Care and Use of Laboratory Animals, with approval from the Institutional Animal Care and Use Committee of the IMB, CAMS (approval number: WSP 201803014).

### Cell Culture

The Vero African green monkey kidney cell line (ATCC, Manassas, Virginia, USA) and the KMB17 cell line (IMB, CAMS, Yunnan, China) were cultured in minimum Eagle’s medium (MEM; Thermo Fisher Scientific, Waltham, Massachusetts, USA) supplemented with 10% fetal bovine serum (FBS; HyClone, GE Healthcare, Chicago, Illinois, USA), 10% 100 U/mL penicillin and 100 mg/mL streptomycin. JASWII dendritic cells (ATCC, Manassas, Virginia, USA) were cultured in MEM Alpha (Thermo FisherScientific, Waltham, Massachusetts, USA) supplemented with 20% fetal bovine serum (FBS; HyClone, GE Healthcare, Chicago, Illinois, USA), 10% 100 U/mL penicillin and 100 mg/mL streptomycin, and 5 ng/mL murine GM-CSF (HY-P7361, MCE, USA), and the cells were maintained at 37°C with 5% CO_2_. The culture medium was changed to MEM (MEM Alpha) supplemented with 2% FBS after viral infection.

### Virus

The HSV-1 wild-type (WT) strain 8F (23) and the HSV-1 mutant strain M6 (22) were used in the experiments. The mutants were verified by PCR and sequencing of PCR products.

### Animal Experiment Design

Balb/c mice were randomly divided into three groups (**Figure S1**).

In group A, the mice were infected with a low dose (2×10^4^ PFU) of the wild-type (WT) or attenuated M6 strain *via* the intranasal route. The mice were observed and weighed every day. The nose, brain, spinal cord, trigeminal ganglion, and inguinal lymph nodes were obtained at 3, 7, 14, 21, and 28 days after viral infection (dpi), followed by viral load detection. At 14, 21, and 28 days post-infection, the blood was obtained for neutralizing antibody testing, and the spleen was subjected to lymphocyte separation for ELISpot assays. At 28 days post-infection, all infected mice were challenged with a lethal dose (1×10^5^ PFU) of the WT strain *via* the intranasal route and observed and weighed every day.

In group B, the mice were infected with 1×10^5^ PFU of the WT or M6 strain *via* the intranasal (IN) route, intradermal (ID) route and intramuscular (IM) route. At 36, 48, and 72 hours post-infection (hpi), tissues (nose, muscle, and skin) from the infected site were obtained for immunofluorescence detection, viral load detection, and qRT-PCR cytokine detection.

In group C, for the transfusion experiment, CD11c^+^ DC cells were obtained from healthy mice’s skin and infected with WT or M6 (MOI=1) *in vitro*. At 24 hpi, the *in vitro*-infected CD11c^+^ DC cells (1×10^5^ cells per mouse), washed with PBS three times, were transfused into 100 healthy mice through the tail vein, respectively. Then, the mice were observed and weighed daily. The spleen, brain, spinal cord, trigeminal ganglion, and inguinal lymph nodes were obtained at 1, 3, 7, 14, 21, 28, and 56 days after adoptive transfer, followed by viral load detection, and at 1, 3, and 7 days inguinal lymph nodes were obtained to detect changes in cytokine levels. At 21 and 28 days after adoptive transfer, the spleen was subjected to lymphocyte separation for ELISopt assays. At 28 days post-transfused, 25 infected mice in each group were challenged with a lethal dose (1×10^5^ PFU) of the WT strain *via* the intranasal route and observed and weighed every day. At 28 days after the challenge, blood was obtained for neutralizing antibody testing.

### Virus Infection of JASWII-Dendritic Cells

Virus-infected JASWII-dendritic cells (or HVEM-specific antibody blocked, rabbit anti-TNFSF14 antibody, BS-2462R) infected with the wild-type strain or attenuated M6 strain were tested for virus proliferation at 8 to 72 h at a MOI=0.1; after infected 12, 24, 36, and 48 h, the cells were used to detect cytokines.

### RT–PCR and qRT–PCR

The viral loads in the tissues were determined by qPCR with absolute quantification. Based on the methods performed by Ryncarz AJ et al. (24), the binding site designed by the primer was located in the HSV-1 gG gene region, and the gG gene was constructed on the p-GMT plasmid as a standard DNA sample. The primers were F: TCCTSGTTCCTMACKGCCTCCC and R: GCAGICAYACGTAACGCACGCT. Viral genomic DNA was extracted from tissues using an AxyPrep™ Body Fluid Viral DNA/RNA Miniprep kit (Central Avenue Union City, CA, USA). The TaqMan probe (Sangon Biotech, Shanghai, China) had the sequence 5’-6FAMCGTCTGGACCAACCGCCACACAGGTTAMRA. The reactions were performed using Premix Ex Taq™ (Probe qPCR; TaKaRa, Dalian, China) on a CFX96 Connect Real-Time System (Bio-Rad, Hercules, CA, USA).

For the relative expression of cytokines in tissues and cells, total RNA was extracted with TRIzol-A+ Reagent (Cat# DP421, Tiangen) according to the manufacturer’s protocol. Gene expression was expressed as the fold-change (2^−ΔΔCt^) relative to the levels in samples from PBS-injected mice or virus-uninfected cells used for calibration. The reactions were performed using a One-Step SYBR Prime Script™ PLUS RT-PCR kit (TaKaRa, Dalian, China). The specific primers used are listed in **Table S1**.

### Virus Titration

The virus titer was determined following standard protocols, as described previously (25). In brief, the virus was subjected to gradient dilution and added to 96-well plates containing 10^4^ Vero cells in each well, and the cells were cultured at 37°C with 5% CO_2_ for 7 days.

### Neutralization Assay

A neutralization assay was performed following standard protocols. Briefly, the diluted serum (1:4, 1:8, 1:16, 1:32, and 1:64) and the virus were mixed with a titer of 100 times the 50% cell culture infectious dose (CCID_50_)/100 μL and incubated at 37°C for 2 hours. Vero cells were then added to 96-well plates and incubated at 37°C, and the CPE of the virus was observed after 1 week.

### IFN-γ-Specific and IL-4-Specific ELISpot ASSAY

The spleen was isolated under sterile conditions, and the splenic lymphocytes were divided into lymphocyte suspensions according to the instructions of the lymphocyte separation solution (Dakewe Biotech, Beijing, China). For the ELISpot assay, the mouse IFN-γ (or IL-4) ELISpot kit (MABTECH Inc., Cincinnati, OH, USA) was used for the manufacturer’s protocol. Briefly, a plate was conditioned and seeded with splenic lymphocytes (10^4^ cells per well) before adding 10 μg of stimulant (two peptides: gB498-505, SSIEFARL; and ICP6 822-829, QTFDFGRL) (Sangon Biotech, Shanghai, China). Then, the cells were incubated at 37°C for 30 h. After that, the cells and medium were removed, and the plate was developed. The colored spots were counted using an automated ELISpot reader (CTL, Cleveland, OH, USA) (22).

### Immunofluorescence and Confocal Microscopy

Skin, nose, and muscle tissue from immunized mice were collected and immediately frozen in liquid nitrogen. According to the manufacturer, the tissue sections were embedded in OCT (Tissue-Tek OCT Compound 4583, Sakura) and sliced on a cryostat at a 5 µm thickness (CM1850, Leica) protocol. The tissue sections were fixed with 4% paraformaldehyde solution for 20 min and blocked with 5% bovine serum albumin (BSA) at 37°C for 2 h. The sections were sequentially incubated with a primary rabbit anti-HSV-1 antibody (Thermo) at 4°C overnight, washed three times with TBST buffer (0.15 M NaCl, 20 mMTris-HCl, 0.05% Tween 20, pH 7.4), and then incubated an Alexa Fluor 647-conjugated donkey anti-rabbit IgG secondary antibody (Invitrogen) at 37°C for 0.5 h to detect the viral antigen. DCs were detected with a rat anti-CD11c antibody (Abcam, Cat# ab33483) and Alexa Fluor 488-conjugated donkey anti-rat IgG secondary antibody (Invitrogen). All cell nuclei were detected with DAPI. Fluorescence was visualized and analyzed using a confocal microscope (TCS SP2, Leica).

### Isolation of Lymphocytes From Mouse Skin

Back skin tissues dissected from euthanized mice were placed into dishes and cut into 0.2mm^2^ pieces with scissors. The pieces of tissue were transferred into 100 mm dishes and incubated with a digestion solution containing 2.5 mg/mL collagenase I (Cat # C0130, Sigma), 2.5 mg/mL trypsin (Cat # 27250018, Thermo Fisher Scientific), and 1 U/mL DNase I (D8071, Solarbio) in Roswell Park Memorial Institute (RPMI) 1640 medium for 2 h at 37°C. The digested supernatant was filtered through a 70 μm cell strainer to obtain a single-cell suspension. Skin lymphocytes were isolated by centrifugation in lymphocyte separation solution (Dakewe Biotech, Beijing, China). CD11c-positive dendritic cells were enriched with the EasySep Mouse CD11c Positive Selection Kit (Stemcell, Cat# 18780) for subtype sorting.

### Bone Marrow-Derived Dendritic Cells

Murine bone marrow (BM) cultures were initiated at 1×10^6^ cells/mL in Roswell Park Memorial Institute (RPMI) 1640 complete medium (Cat#R8758, Sigma) containing 20 ng/mL GM-CSF (HY-P7361, MCE, USA) and 10 ng/mL IL-4 (HY-P70644, MCE, USA). Cells were collected following 6-7 days of culture.

### Flow Cytometry Analysis and Cell Sorting

Skin and spleen lymphocytes were cultured in Roswell Park Memorial Institute (RPMI) 1640 medium (Cat #R8758, Sigma) supplemented with 10% fetal bovine serum (FBS; HyClone, GE Healthcare, Chicago, Illinois, USA), 10% 100 U/mL penicillin and 100 mg/mL streptomycin, and the cells were maintained at 37°C with 5% CO_2_. To sort the pDCs subgroup, first use the B cell positive selection kit to isolate B cells (EasySep™ Mouse CD19 Positive Selection Kit, Catalog #18754). Then, the liquid passing through the column of B cell positive selection was performed by EasySep Mouse CD11c Positive Selection Kit (Stemcell, Cat# 18780) to enrich DCs cells. The CD11c-positive DCs were washed three times with PBS, and then added 10μL fluorophore-conjugated antibody (PerCP/Cy5.5-CD103 (Cat#121416), PE/Cy7-CD11c (Cat#117318), FITC-CD11b (Cat#101206), PE-CD45R (Cat#103208) and APC-CD8α (Cat#100712) purchased from BioLegend). The cells were stained for 30 min at 4°C and washed twice prior to flow cytometric analysis (LSR Fortessa, BD) and to sort (Influx, BD).

### TranscriptomeAnalysis of Dendritic Cells

The skin CD103^+^ dendritic cells were sorted by flow cytometry and infected with WT and M6 (MOI=0.1) at 24, 48, and 72hpi. At the same time, a control group (CD103^+^ DCs) without viral infection was established. According to the manufacturer’s instructions, total RNA was extracted using TRIzol (Cat # DP421, Tiangen). RNA quantity and integrity were evaluated using the NanoDrop system and a Bioanalyzer, and the samples were prepared according to Illumina’s instructions and sequenced (Gene Denovo Biotechnology Co., Guangzhou, China). Genes with 2-fold or greater changes in expression at *P*< 0.05 in the Kyoto Encyclopedia of Genes and Genomes (KEGG) analyses were selected and grouped into functional categories. The raw sequence data were deposited in the Sequence Read Archive under BioProject number PRJNA834578.

### Statistical Analysis

All the data are expressed as the mean value with the standard error of the mean. Significant differences between groups were analyzed by two-way ANOVA (GraphPad Prism; GraphPad Software, San Diego, CA, USA), and *P*< 0.05 was considered to indicate statistical significance.

## Results

### The Attenuated HSV-1 Mutant Strain M6 Leads to an Effectual Immune Response in Comparison With the Wild-Type Strain

**Figure 1:**
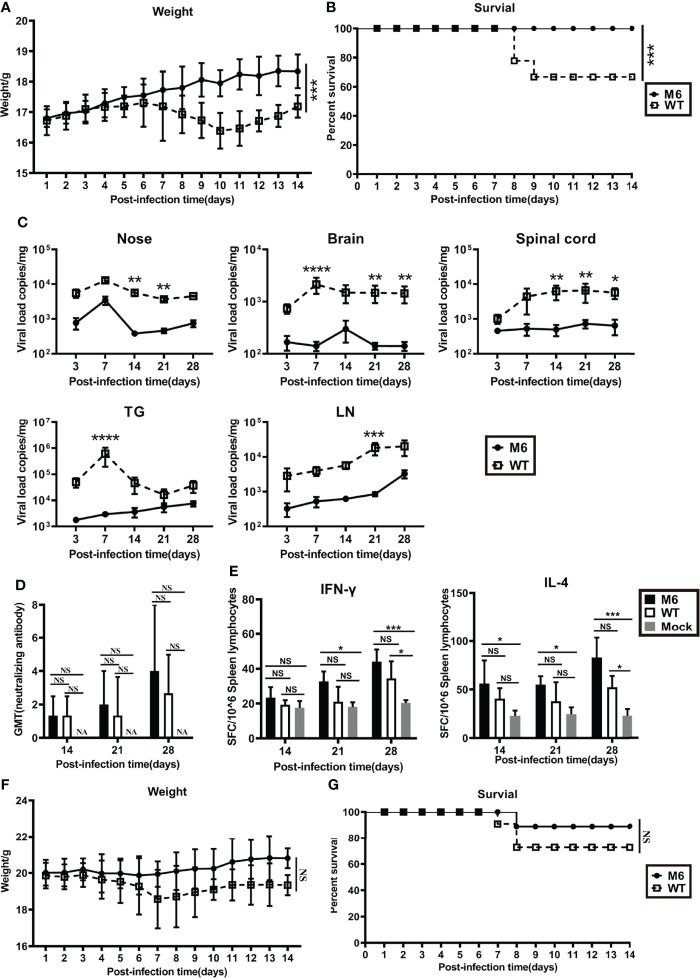
The mutated strain M6 produced a more effective immune response than the wild-type strain. Bodyweight **(A)** and survival rate **(B)** of mice infected with the attenuated HSV-1 mutant M6 (circle) and the wild-type strain (square) *via* nasal spray. **(C)** Viral loads in the nose, brain, spinal cord, trigeminal ganglion, and inguinal lymph node after infection with M6 (circle) and WT (square), as determined by RT–qPCR. **(D)** The HSV-1 neutralizing antibody titer in mice infected with M6 (black square), WT (white square), and Mock (gray square). **(E)** The ELISpot responses show IFN-γ- and IL-4-secreting cells among splenic lymphocytes after infection with M6 (black square), WT (white square), and Mock (gray square). Bodyweight **(F)** and survival rate **(G)** of mice immunized with M6 (circle) and WT (square) after wild-type viral challenge. The data are shown as the mean ± SEM based on data from three independent experiments. *P < 0.05, **P < 0.01, ***P < 0.001, ****P < 0.0001. NS, no significance.

Our previous published work showed that this M6 strain was capable of leading to an immunoprotective effect after inoculation in mice *via* nasal spray, with indicators of neutralizing antibodies, specific T cell responses with IFN-γ and IL-4 production 4 to 8 weeks after inoculation in the mice, and clinical protection, including a lower viral load in tissues and mild pathologic injuries observed during virus challenge (22). Here, we further compared the characteristics of the immune responses and pathologic injuries elicited by the M6 and wild-type strains in Balb/c mice and found first that mild clinical manifestations, including weight loss and death rate, were observed in the M6 group compared to the wild-type strain group (**Figures 1A, B**); second, the viral load assay in various tissues from sacrificed animals at different times post-inoculation did not show obvious proliferative peaks in the M6 group (**Figure 1C**); and third, the neutralizing antibody assay and ELISpot detection of specific T cell responses of mice showed that the M6 strain elicited a no less strong response than by the wild-type strain (**Figures 1D, E**). Further observation of the mice inoculated with the M6 or wild-type strain in the viral challenge test suggested different clinical outcomes in the two groups (**Figures 1F, G**), with decreased body weight and a higher death rate in the group inoculated with the wild-type strain. These results identified the M6 strain as an attenuated strain that induces mild pathologic injury in mice and better immunogenicity, eliciting an immunoprotective effect. This result is significant compared to the wild-type strain for investigating the relationship between HSV-1 infection in dendritic cells and the development of antiviral immunity.

### Differences in Infection Between the Wild-Type Strain and Attenuated M6 Strain in Cultured Mouse JAWSII-Dendritic Cells

**Figure 2:**
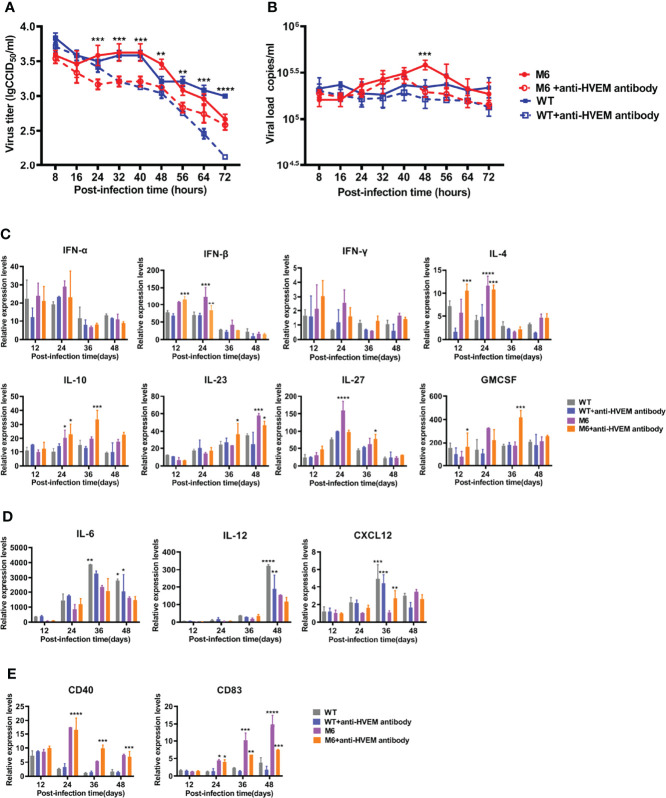
The dynamic processes in JAWS II-dendritic cells infected with the wild-type strain and attenuated M6 strain. The replication **(A)** and viral load **(B)** of JASWII-dendritic cells infected with the wild-type strain (solid blue square and hollow square) and attenuated M6 strain (solid red circle and hollow circle). The M6+anti-HVEM antibody group is compared with the M6 group, and the WT+anti-HVEM antibody group is compared with the WT group. Transcriptional profile analysis of immune regulators and effectors **(C, D)** in JASWII-dendritic cells infected with the wild-type strain (gray and blue) and attenuated M6 strain (purple and orange). The relative expression levels of inflammatory cytokines in JAWS II dendritic cells were normalized to their levels in the blank control group (without viral infection and antibody) using the comparative Ct (ΔΔCt) method. The data are shown as the mean ± SEM based on data from three independent experiments. *P < 0.05, **P < 0.01, ***P < 0.001, ****P < 0.0001.

Based on the disparate immunological phenotypes observed in mice infected with the wild-type strain and attenuated M6 strain, our work using cultured JAWS II-dendritic cells investigated the dynamic infection process with the two virus strains. The results indicated that the wild-type strain or the attenuated strain replicated in these dendritic cells with different efficiencies (**Figure 2A**), while the viral proliferation of both strains was inhibited in the cells treated with a specific antibody against HVEM (**Figure 2A**). Other viral load detection in these infected JAWS II-dendritic cells confirmed this finding (**Figure 2B**). The transcriptional profile analysis of some immune regulators and effectors in these infected cells *via* q-RT–PCR found that most immune molecules, including IFN-α, IFN-β, IFN-γ, IL-4, IL-10, IL-23, IL-27, and GMCSF, which are important for the activation of antiviral immunity, were consistently upregulated in the infected cells by the attenuated strain (**Figure 2C**), while the expression of marker molecules for the maturation of dendritic cells, such as CD83 and CD40, are less activated in WT group than in M6 group (**Figure 2E**). However, no significant variation in these molecules was found in cells infected with the wild-type strain (**Figures 2C, D**). Interestingly, some inflammatory factors, such as IL-6, IL-12, and CXCL12, were upregulated in cells infected with the wild-type strain (**Figure 2D**). These results suggested the capacity of HSV-1 to infect certain dendritic cells and that dendritic cells respond differently to viral infection depending upon the virulence of the viral strain. Theoretically, this difference might impact the phenotype of antiviral immunity.

### HSV-1 Infection in Dendritic Cells Alters the Dynamic Process of the Innate Immune Response in Local Tissue

**Figure 3:**
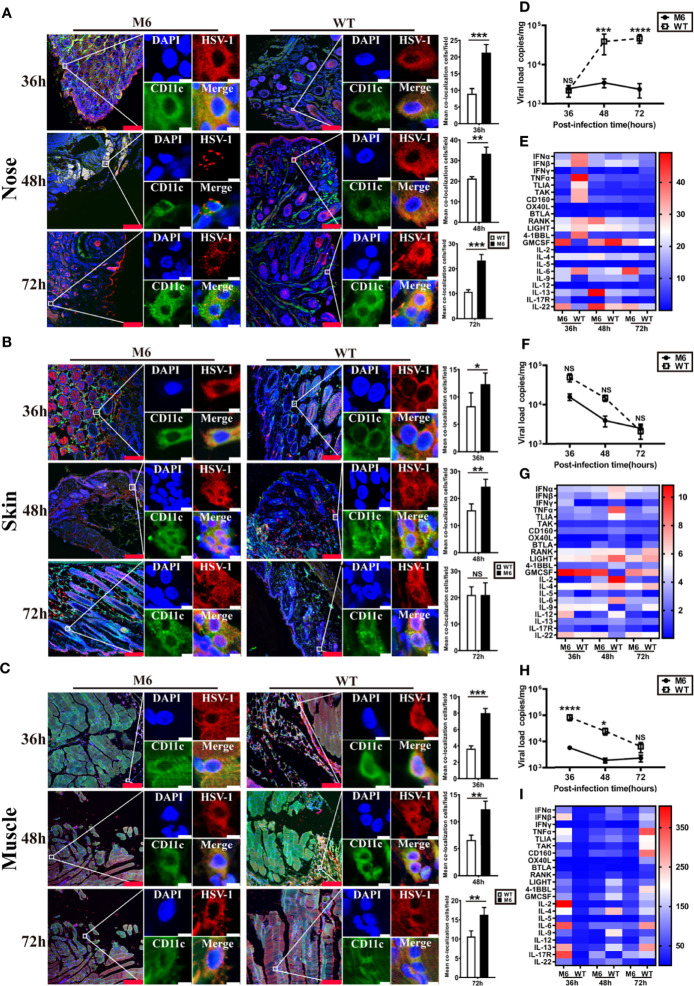
The innate immune responses in local tissues after HSV-1 *via* different infection routes. Representative confocal fluorescence images of HSV-1 expression (red) and CD11c (green) after intranasal (IN) **(A)**, intradermal (ID) **(B)** and intramuscular (IM) **(C)** administration of HSV-1 antigen at 36 h, 48 h and 72 hpi. The image on the right represents the colocalization rates of DC cells after intranasal (IN) **(A)**, intradermal (ID) **(B)**, and intramuscular (IM) **(C)** administration. White represents M6 infection, and black represents WT infection. Representative fluorescent cells in the white rectangle are shown at 20× magnification. The red scale bar is 100 µm, white scale bar is 5 µm. Statistical analysis of colocalization with both HSV-1 and DCs (marked by CD11c) compared to observations of 50 DCs randomly counted. Viral load in the nose **(D)**, skin **(F)**, and muscle **(H)** tissue of mice infected with M6 (circle) and WT (square) *via* different routes. Expression profiles of immune regulatory molecules in the nose **(E)**, skin **(G)**, and muscle **(I)** tissue of mice infected with M6 and WT. A heat map shows the dynamic gene expression profiles. Each column represents one dataset. The red color indicates the genes expressed at higher levels in the samples than those at 0 hpi, and the blue color indicates the genes expressed at lower levels. The darker the color, the more significant the change. The data are shown as the mean ± SEM based on data from three independent experiments. *P < 0.05, **P < 0.01, ***P < 0.001, ****P < 0.0001. NS, no significance.

Based upon the observed characteristics of the immune response elicited by the attenuated M6 strain and the biological process of M6 infection in cultured dendritic cells, our work investigated the interaction of this strain or the wild-type strain and dendritic cells in infected mice and suggested interesting differences in the mouse groups inoculated with the two strains at the same infectious dose (10^5^ CCID_50_/mouse). Observation of the nasal mucosa, intradermal and muscular tissues using a fluorescence microscope indicated that the attenuated M6 strain had a higher rate of antigenic colocalization with dendritic cells in three tissues at 36, 48, and 72 hours post-inoculation than the wild-type strain (**Figures 3A–C**). The viral load of the M6 strain in these infected tissues was lower than that of the wild-type strain in these tissues (**Figures 3D, F, H**). These results suggest that wild-type strain infection might attenuate the phagocytosis of viral antigen by dendritic cells in epithelial tissues *via* some unknown mechanisms. mRNA transcriptional profile assays of various immune-regulating molecules in these three tissues suggested that the two strains led to different profiles (**Figures 3E, G, I**), with some important molecules, including type 1 interferon (including IFN-α, IFN-β), IL-6 and TNF-α, showing significant differences in all tissues between the two groups (**Figures 3E, G, I**). The difference was significant in the nasal mucosa (**Figure 3E**). These results indicate that the characterized interaction of the HSV-1 wild-type strain and dendritic cells might be involved in the varied innate immune responses and inflammatory reactions in tissues, which might be relevant to specific antiviral immunity.

### Infection With a Wild-Type Strain and Attenuated Strain in Dendritic Cells Leads to the Differentiation States With Disparate Biological Characteristics

**Figure 4:**
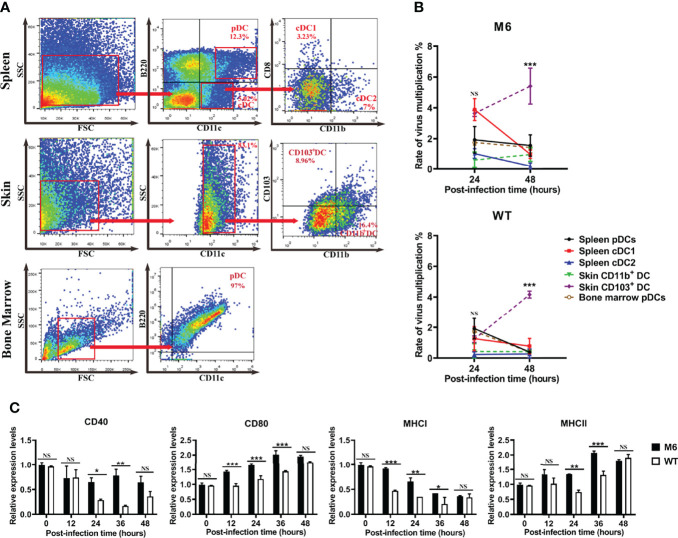
M6- and WT-infected dendritic cells. **(A)** Flow cytometric sorting of dendritic cells from different tissues. **(B)** Rate of virus multiplication in dendritic cells with different surface markers after M6 and WT infection ((the viral load (24 or 48 h)-the viral load (0 h))/the viral load (0 h)). The black circle represents the data in spleen pDCs infected with the virus, the red square represents those in infected-spleen-cDC1, the blue triangle represents those in infected-spleen-cDC2, the green triangle represents those in infected-skin-CD103^+^ DC, the purple circle represents those in infected-skin-CD11b^+^ DC, and the brown circle represents those in infected-bone-marrow-pDCs. **(C)** mRNA expressions of key surface markers on dendritic cells infected with M6 (circle) and WT (square). The relative expression levels of key surface markers in dendritic cells were normalized to their levels in the blank control group (without viral infection) using the comparative Ct (ΔΔCt) method. The data are shown as the mean ± SEM based on data from three independent experiments. *P < 0.05, **P < 0.01, ***P < 0.001, ****P< 0.0001. NS, no significance.

The wild-type strain and attenuated strain are capable of infecting dendritic cells, and the available information about the immunological function of dendritic cells suggests the significance of the interaction between viral infection and various subpopulations of dendritic cells for understanding the immune response after HSV-1 infection. Dendritic cells with different surface markers were collected from normal Balb/c mice and cultured *in vitro.* Furthermore, these cells were infected with the wild-type strain or attenuated strain, and their surface marker molecules were detected at different time points of 24 and 48 hpi. The results suggested that both strains can not only infect plasmacytoid dendritic cells and conventional dendritic cells but also stimulate them to further differentiate to a state with a CD103^+^ phenotype (**Figures 4A, B**). Interestingly, the transcriptional profiles of some significant immune molecules of these infected CD103^+^ dendritic cells suggested that the cells infected with the attenuated M6 strain showed higher expression of the immune molecules than those in cells infected with the wild-type strain (**Figure 4C** and **Figure S2**), which seems to indicate that infection with the wild-type strain inhibits cellular immune activity, as these cells are involved in antigen presentation to T cells. Previous data showed that CD103^+^ dendritic cells possess powerful antigen presentation capacity (26). All of the data from our experiment suggested different interactions between the wild-type strain and attenuated strain, which implies a possible mechanism for the ineffective immune response in the infected population.

### Viral Infection in CD103^+^ Dendritic Cells Leads to Alteration of the Transcription Profile Related to the Immune Response

**Figure 5:**
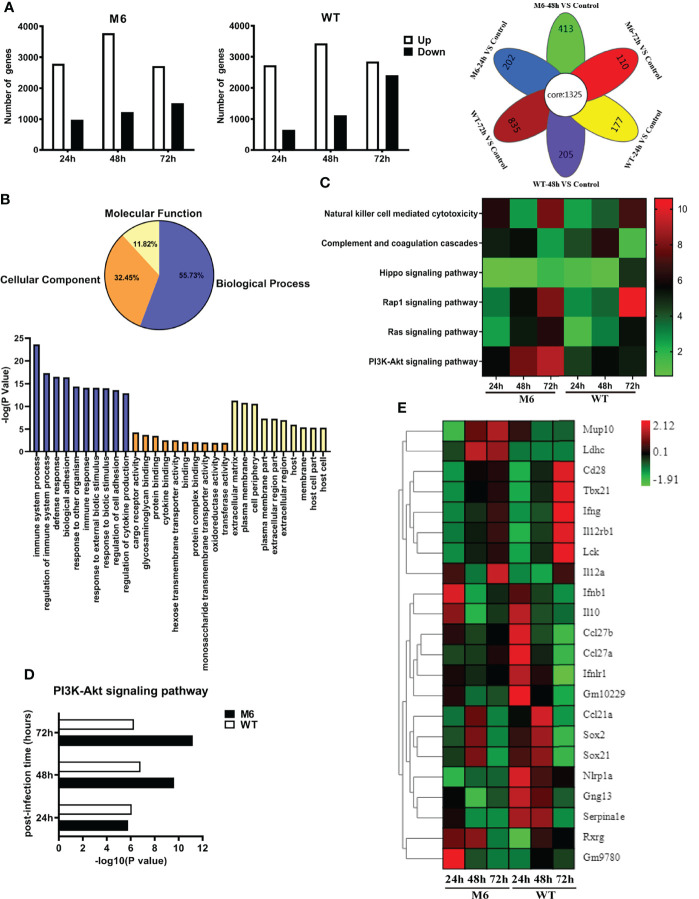
The change in the transcriptional profile of CD103^+^ dendritic cells infected with the wild-type strain and attenuated M6 strain. **(A)** The number of significantly differential genes in dendritic cells infected with M6 and WT. All the data of infected samples were compared with those of the control group (blank DCs without viral infection). The white square represents upregulated genes, and the black represents downregulated genes. The image on the right shows the Venn diagram of the differential genes of M6 and WT infection DCs at different times. The overlapping differentially expressed genes are shown as “core” between the M6-infected and WT-infected groups during the entire sampling period (24, 48, and 72 hpi). **(B)** Gene Ontology enrichment terms for differential genes in dendritic cells infected with M6 and WT, compared with the control group. Blue represents the analysis of biological progress, light yellow represents the analysis of molecular function, and orange represents the cellular component analysis. The histograms represent the number of GOs annotated as unique GO terms and presented in the below panel. **(C)** The pathways involved in immune-related differential gene expression for M6 and WT compared with the control group. Each row represents a pathway, and the samples are depicted in the columns. Red indicates the numbers of the differentially expressed genes in the more pathways, and green denotes those of the differentially expressed genes in the pathways that were less than the control. **(D)** Compared with the control, the number of significant differential genes involved in PI3k-Akt pathways after M6 and WT infection of dendritic cells. **(E)** Fold changes in the expression of differential genes in infected dendritic cells at different times compared with the control. A heat map and supervised hierarchical clustering analysis revealed 22 genes associated with T and B cells activation. Each row represents a gene, and the samples are depicted in the columns. Red indicates genes expressed at higher levels, and green denotes genes expressed at lower levels. The color bars represent log 2 of fold change.

Based upon the characterized biological features of dendritic cells infected by the HSV-1 wild-type strain or attenuated strain, we analyzed the dynamic alterations in the mRNA profile of infected CD103^+^ cells at different time points after infection. The results suggested different alterations within the two groups and showed a consistently increasing number of upregulated differential genes in the two groups during the infectious process (**Figure 5A**). These data also suggested that the wild-type strain altered the transcriptional profile based upon its pathogenic effect on dendritic cells, even though its proliferation peak was lower than that of the attenuated M6 strain. The analysis suggested that these different genes were related to various cellular biological properties (**Figure 5B**), especially genes that play a role in immune regulation (**Figure 5B**). Further analysis found that the expression of genes related to cytotoxic effects mediated by NK cells, complement reactions, and the signaling pathways of Rap1, PI3K-Akt, and Hippo presented different patterns of alterations in dendritic cells infected with the M6 strain and wild-type strain (**Figure 5C**). Among these genes with varied expression, the abundance of genes related to the PI3K-Akt signaling pathway in the cells was upregulated gradually starting at 24 hours post-infection by the M6 strain and reached the highest degree of variation (**Figure 5D**), while this tendency was reversed in cells infected with the wild-type strain (**Figure 5D**). The differences in expressed genes between the groups were principally involved in T and B cells activation (**Figure 5E**), including CD28, Lck, and Tbx21.

### Information Carried in Dendritic Cells Infected With HSV-1 is Insufficient to Establish Effective Antiviral Immunity

**Figure 6:**
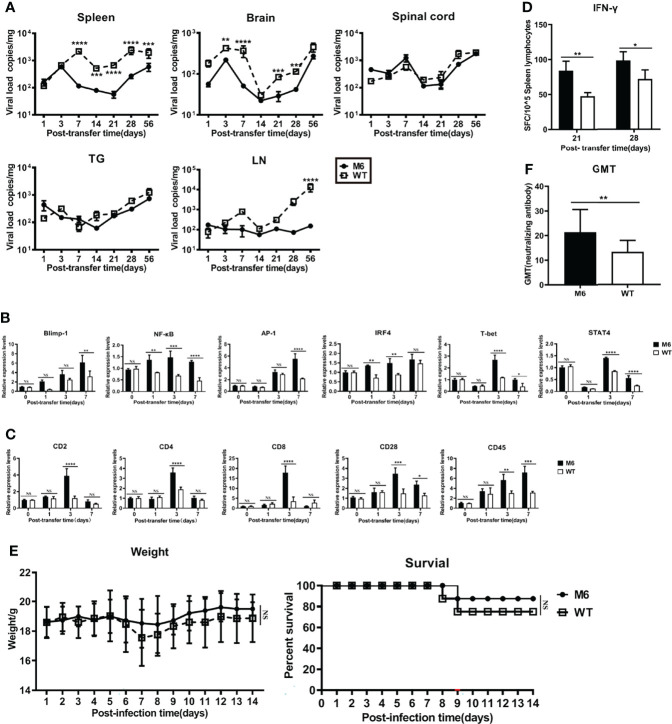
Dendritic cells carrying HSV-1 antigen were adoptively transferred into mice. **(A)** As determined by RT-qPCR, viral load in the spleen, brain, spinal cord, trigeminal ganglion, and inguinal lymph node after DCs carrying M6 (circle) and WT (square) antigens transferred into mice. **(B)** The expression levels of genes associated with T-cell activation in inguinal lymph node tissue after transferring dendritic cells infected with WT (white square) and M6 (black square) compared with the control group (transferring uninfected DCs). Detection of the p105 subunit to represent the level of NF-κB and detection of the c-Jun subunit to represent the level of AP-1. The results are normalized by the endogenous GAPDH expression level. The expression levels were calculated using the comparative Ct (ΔΔCt) method by relative quantification. **(C)** The expression levels of genes associated with surface marker molecules after T cell activation in lymph node tissue after transferring dendritic cells infected with WT (white square) and M6 (black square) compared with the control group. The results are normalized by the endogenous GAPDH expression level. The expression levels were calculated using the comparative Ct (ΔΔCt) method by relative quantification. **(D)** The ELISpot responses show IFN-γ-secreting T cells among splenic lymphocytes after transferring dendritic cells carrying M6 (black square) and WT (white square) antigens. **(E)** Bodyweight and survival rate of mice subjected to transfer of dendritic cells carrying M6 (circle) and WT (square) antigens followed by wild-type challenge. **(F)** The titer of neutralizing antibody against HSV-1in transferred-DCs mice after being challenged with the wild-type strain. The samples were obtained at 28 dpi. The data are shown as the mean ± SEM based on data from three independent experiments. *P< 0.05, **P < 0.01, ***P < 0.001, ****P < 0.0001. NS, no significance.

The above data revealed the immunologic features of dendritic cells infected with the HSV-1 wild-type strain and suggested the physiological role of dendritic cells, which enables their uptake of viral antigens and their transfer to lymph nodes for antigen presentation to T cells, might be modified to some extent. To investigate this possibility, we infected dendritic cells (CD11c) isolated from the skin of mice with the wild-type strain or attenuated M6 strain and transferred them to mice intravenously 24 hpi. The evaluation of these mice suggested that the viral load in some tissues of mice receiving dendritic cells infected with the wild-type strain 4 weeks after the transfer was higher than that of mice receiving cells infected with the M6 strain (**Figure 6A**), especially in the inguinal lymph nodes (**Figure 6A**). The detection of mRNA transcripts related to immune regulation in lymph node tissue revealed different expression profiles of genes related to T-cell activation in both groups (**Figure 6B**), and the expression of genes encoding marker molecules on the activated T-cell surface presented different trends in both groups (**Figure 6C**). Further IFN-γ-specific ELISpot assays against the viral gB protein to evaluate the T cell response showed higher counts in the M6 strain group than in the wild-type strain group (**Figure 6D**). In a viral challenge test *via* the nasal route, the two groups of mice receiving dendritic cells infected with the wild-type strain or the M6 strain showed obvious differences in body weight and death rate (**Figure 6E**), suggesting different capacities of immune defense against virus infection in the two groups of mice, which was supported by the different neutralizing antibody GMT values of the two groups (**Figure 6F**).

## Discussion

Studies of the immune response in individuals infected with HSV-1 suggested the low efficacy of immune-mediated viral clearance *in vivo* and defense against viral reinfection due to the interference of various virus-encoded proteins in innate and adaptive immunity (27–29), leading to challenges in developing antiviral drugs and vaccines and highlights the need to elucidate the detailed mechanism of viral interference with the immune system. Both HSV-1 and HSV-2 were found to be able to infect dendritic cells due to membrane glycoprotein D binding to the HVEM receptor (30–33). This receptor, as a member of the TNF receptor family, can interact with the LIGHT molecule with the assistance of TNF-α from NK cells activated by IL-2 secreted from local tissues during inflammatory reactions (34, 35) and is involved in the differentiation and maturation of dendritic cells (34). During HSV-1 infection, this physiological process might be utilized to lead to not only the active phagocytosis of antigen from infected epithelial tissue by dendritic cells but also dendritic cell infection by the virus *via* gD protein binding to HVEM, which could be recognized as one viral strategy to disturb the host immune system (36). These data raised the question of what outcome could be induced by these infected dendritic cells in innate and adaptive immunity. M6, an attenuated strain, was used in our study since it could elicit significant immune protection in mice (22). We analyzed HSV-1 infection in mouse JAWS II-dendritic cells and the subsequent variations in the cellular immune phenotype. Furthermore, our study in mice investigated the variations in the innate immune response and inflammatory response induced by the wild-type strain and attenuated strain during infection of dendritic cells in local tissues and outlined some events induced by these variations during the process viral infection in detail. We aimed to elucidate the immunological mechanism occurring in individuals infected with HSV-1 who cannot eliminate the virus *in vivo* post-infection or prevent reinfection by comparing infection with wild-type and attenuated strains in dendritic cells. Our work not only identified HSV-1 infection in dendritic cells with the surface markers CD11c and CD11b *in vitro* and *in vivo* but also revealed that this infection enabled the stimulation of cellular differentiation to the CD103^+^ phenotype, which was described as being associated with a powerful capacity for antigen presentation (26). Interestingly, CD103^+^ cells infected with the wild-type and attenuated strains showed different transcriptional profiles of most immune regulators and effector molecules. Considering the efficiency of antiviral immunity elicited by the attenuated strain in animals, these results implied a strategy by which the HSV-1 wild-type strain interferes with the development of effective antiviral immunity, which is probably due to viral replication and associated with some viral-encoded proteins that modify the immunological phenotype of dendritic cells during antigen processing and presentation to T cells. Specific activation of T cells depends on the ability to accurately transfer antigenic information carried in dendritic cells to the T-cell population (37), and a small error in this process might lead to disruption of the immune response. The results obtained in our experiment on dendritic cells infected with the wild-type strain and attenuated strain and transferred to normal mice further suggested that the antigen information carried in infected cells by the wild-type strain did not work well in the recipient mice in comparison to that from cells infected with the attenuated strain.

All of the data support the conclusion that HSV-1 infection in dendritic cells based upon envelope glycoprotein D binding to HVEM is an important component of the viral infection strategy, with alterations in the phenotype of the active response of dendritic cells during recognition of viral antigens and transfer of antigen information to T cells from infected tissues *via* interference with the cellular transcription of various genes. The outcome of this event might lead to a series of deviations in the immune response, with a deficient immune phenotype in infected individuals in the clinic. However, the details of the mechanism need further investigation in the future.

## Data Availability Statement

The datasets presented in this study can be found in online repositories. The name of the repository and accession number can be found below: National Center for Biotechnology Information (NCBI) BioProject, https://www.ncbi.nlm.nih.gov/bioproject/, PRJNA834578.

## Ethics Statement

The animal study was reviewed and approved by The Institutional Animal Care and Use Committee of the IMB, CAMS (approval number: WSP 201803014).

## Author Contributions

QHL and YZ conceived and designed the study; JJZ, XLX, SQD, YG, and DJM performed the research; RY, FYZ, XQL, ZYM, XHL, ZYN, GRJ, and LCW contributed reagents; LY, YL, DDL, and HZ contributed materials; JJZ, QHL, and YZ analyzed the data; QHL wrote the first draft. All authors contributed to the article and approved the submitted version.

## Funding

This work was supported by the National Natural Science Foundation of China (No. 82171817 and No.81802868) and grants from the Major Science and Technology Special Projects of Yunnan Province (No.202001AU070142, No.202002AA100009, No. H-2019060).

## Conflict of Interest

The authors declare that the research was conducted without any commercial or financial relationships construed as a potential conflict of interest.

## Publisher’s Note

All claims expressed in this article are solely those of the authors and do not necessarily represent those of their affiliated organizations, or those of the publisher, the editors and the reviewers. Any product that may be evaluated in this article, or claim that may be made by its manufacturer, is not guaranteed or endorsed by the publisher.
