# Supplementary material for: Characterization of the Immunologic Phenotype of Dendritic Cells Infected With Herpes Simplex Virus 1
Source: Front Immunol. 2022 Jul 5;13:931740. doi: 10.3389/fimmu.2022.931740 (PMC9294641; doi:10.3389/fimmu.2022.931740)
Supplement: Supplementary file 1 [file DataSheet_1.docx]

**
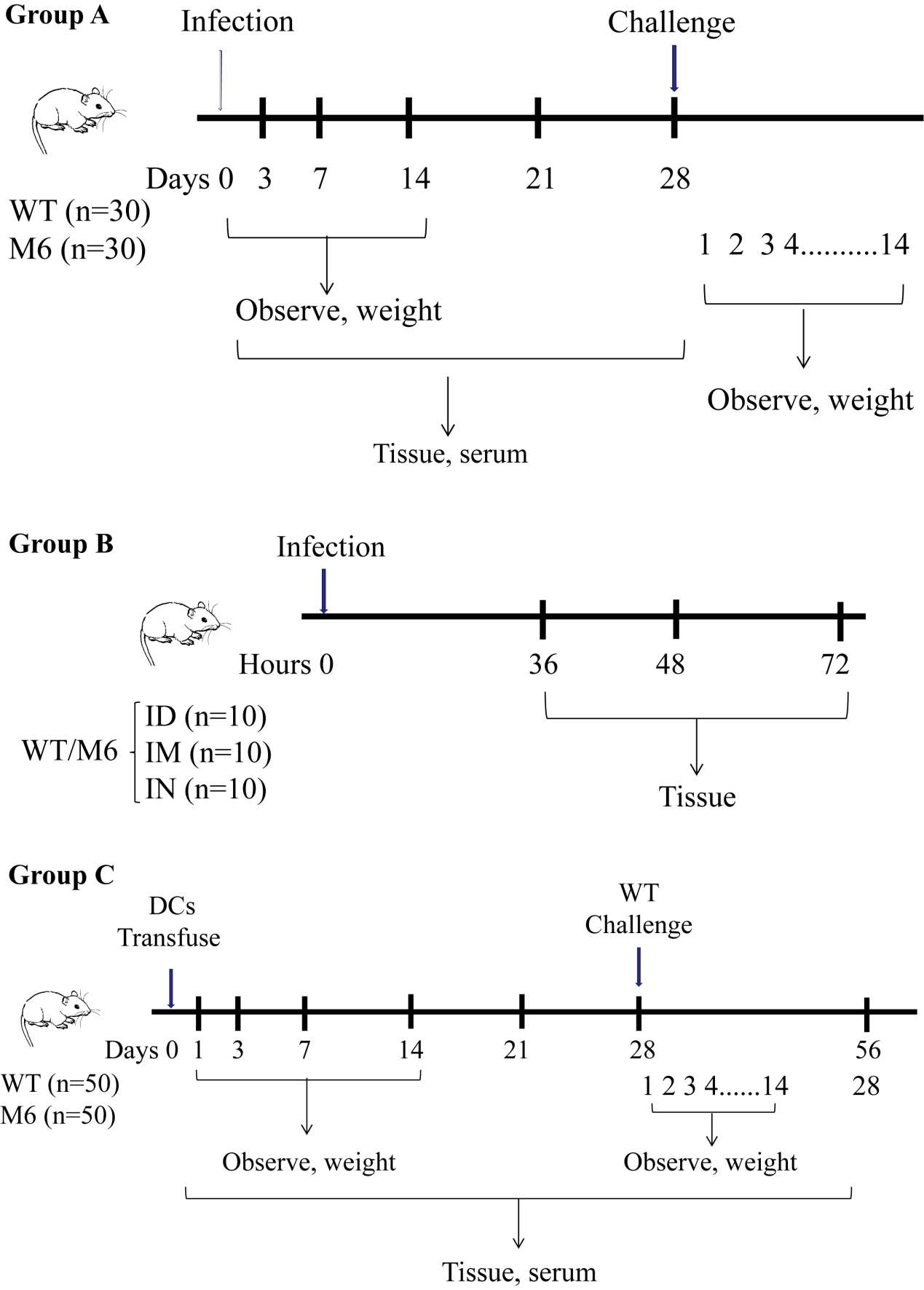
**

**Fig. S1. The design of** [**animal**](javascript:;) [**experiment**](javascript:;)**s.** The animal experiment design of groups A, B, and C were described in Methods. Three mice were euthanized at each point to obtain tissues.


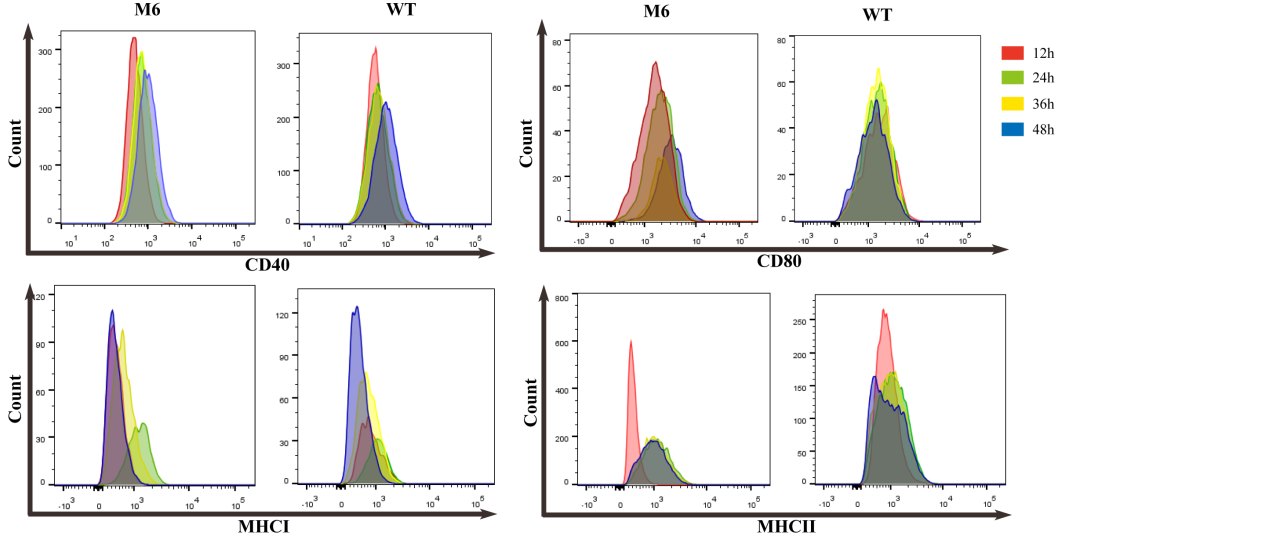


**Fig. S2. The expression of cell surface molecular after WT or M6 infection.** The red represents cells infected for 12 h, green represents cells infected for 24 h, yellow represents cells infected for 36 h, and blue represents cells infected for 48 h.

**Table S1. Primers used for q-RT-PCR**

| Primer | Sequence(5'-3') |
| --- | --- |
| GAPDH-F | AGGTCGGTGTGAACGGATTTG |
| GAPDH-R | TGTAGACCATGTAGTTGAGGTCA |
| IFNα-F | CTTCCTCAGACTCATAACCT |
| IFNα-R | AGTCCTTCCTGTCCTTCA |
| IFNβ-F | GATGAACTCCACCAGCAGACAGTG |
| IFNβ-R | CACCATCCAGGCGTAGCTGTTG |
| IFNγ-F | ATCAGGCCATCAGCAACAACA |
| IFNγ-R | CGTCTCACCTCAAACTTGGCA |
| TNFα-F | GCCAACGGCATGGATCTCAA |
| TNFα-R | TCTTGACGGCAGAGAGGAGG |
| TLIA-F | AATAAGCAACAACTGGTTCC |
| TLIA-R | ATTAGTCTGTCTCCTTCTTCC |
| CD160-F | CCTGAGACCAACTTAGAACA |
| CD160-R | ACACCAACTGAGATGACTT |
| OX40L-F | TGCTTCTGTGCTTCATCTAT |
| OX40L-R | ATCTGGTAACTGCTCCTCT |
| BTLA-F | GCCAGGACAGGAGAGTTA |
| BTLA-R | CTTACACCAAGTCACATTAGG |
| RANKL-F | TCACTCTGTCCTCTTGGTA |
| RANKL-R | CAGGTAATAGAAGCCATCTTG |
| LIGHT-F | TTCTGAGCACCTACATTCC |
| LIGHT-R | CTTCTGACCAACCATTCCT |
| 4-1BBL-F | AAGCCTCAGGTAGATGACT |
| 4-1BBL-R | GAACGGTCCACTAACTTGT |
| GMCSF-F | GGCCTTGGAAGCATGTAGAGG |
| GMCSF-R | GGAGAACTCGTTAGAGACGACTT |
| IL-2-F | GAGCAGGATGGAGAATTACAGG |
| IL-2-R | GTCCAAGTTCATCTTCTAGGCAC |
| IL-4-F | GTGAGCTCGTCTGTAGGGCT |
| IL-4-R | CCGCTTACCGATGAATCCAGG |
| IL-5-F | CTCTGTTGACAAGCAATGAGACG |
| IL-5-R | TCTTCAGTATGTCTAGCCCCTG |
| IL-6-F | TAGTCCTTCCTACCCCAATTTCC |
| IL-6-R | TTGGTCCTTAGCCACTCCTTC |
| IL-9-F | ATGTTGGTGACATACATCCTTGC |
| IL-9-R | GACGGTGGATCATCCTTCAG |
| IL-12-F | CCTCCTGTGGGAGAAGCAGA |
| IL-12-R | CTTGAGCCTTTCAGGCGGAG |
| IL-13-F | CCTCATGGCGCTTTTGTTGAC |
| IL-13-R | TCTGGTTCTGGGTGATGTTGA |
| IL-17R-F | AGATTACTACAACCGATCCACCT |
| IL-17R-R | GGGGACAGAGTTCATGTGGTA |
| IL-22-F | GCTTGACAAGTCCAACTTCCA |
| IL-22-R | GCTCACTCATACTGACTCCGT |
| IL-23a-F | AATAATGTGCCCCGTATCCAGT |
| IL-23a-R | GCTCCCCTTTGAAGATGTCAG |
| IL-27-F | CTGTTGCTGCTACCCTTGCTT |
| IL-27-R | CACTCCTGGCAATCGAGATTC |
| CXCL12-F | CACTGCCTATGTCCTCTTC |
| CXCL12-R | ACTGTTCTCCTGCTCCTT |
| CCL21-F | GTGATGGAGGGGGTCAGGA |
| CCL21-R | GGGATGGGACAGCCTAAACT |
| CD2-F | CAACTTTCAAATGACTGATGATATT |
| CD2-R | TAAGACCTCATACGTTTCTGATATCA |
| CD4-F | AAAGCGGTCATAAGTCAG |
| CD4-R | TCTAAGATAGCATCGGAAA |
| CD8-F | AGACAGTGGAGCTGAAGTGCCAGGT |
| CD8-R | TTTGGGAGAGGTATAGGAGGAAGG |
| CD28-F | TAGATAGCAACGAGGTCAGCCTCAG |
| CD28-R | TCCCATTCCCGACACAGACTTCC |
| CD40L-F | GAGGATCCTCAAATTGCAGCACAC |
| CD40L-R | ATTTTCAAGCATTACCAAGTTGCT |
| CD40-F | TGTCATCTGTGAAAAGGTGGTC |
| CD40-R | ACTGGAGCAGCGGTGTTATG |
| CD45-F | ACTGACCCTCCAAGCACAACCATAGC |
| CD45-R | GAGGTCTGCCTTAAAAGTCTGATT |
| CD80-F | CTGCAAAGGACTTCAGAAACCT |
| CD80-R | AGGCTTCACCTAGAGAACCGT |
| MHCI-F | CCTACCAGAGAATGATTGGCTG |
| MHCI-R | GCAACTCATGCAGGTTGGC |
| MHCII-F | AGTGCGACGAGAACGGTAAC |
| MHCII-R | CGTTGGGGAACACACACCA |
| Blimp-1-F | TTCTCTTGGAAAAACGTGTGGG |
| Blimp-1-R | GGAGCCGGAGCTAGACTTG |
| NF-κB-F | ATGGCAGACGATGATCCCTAC |
| NF-κB-R | TGTTGACAGTGGTATTTCTGGTG |
| AP-1-F | CCTTCTACGACGATGCCCTC |
| AP-1-R | GGTTCAAGGTCATGCTCTGTTT |
| IRF4-F | TCCGACAGTGGTTGATCGAC |
| IRF4-R | CCTCACGATTGTAGTCCTGCTT |
| T-bet-F | AGCAAGGACGGCGAATGTT |
| T-bet-R | GGGTGGACATATAAGCGGTTC |
| STAT1-F | TCACAGTGGTTCGAGCTTCAG |
| STAT1-R | GCAAACGAGACATCATAGGCA |
| STAT4-F | TGGCAACAATTCTGCTTCAAAAC |
| STAT4-R | GAGGTCCCTGGATAGGCATGT |
| Smad2-F | ATGTCGTCCATCTTGCCATTC |
| Smad2-R | AACCGTCCTGTTTTCTTTAGCTT |
| Runx3-F | CAGGTTCAACGACCTTCGATT |
| Runx3-R | GTGGTAGGTAGCCACTTGGG |
| Foxp3-F | CCCATCCCCAGGAGTCTTG |
| Foxp3-R | ACCATGACTAGGGGCACTGTA |
| RoRα-F | GTGGAGACAAATCGTCAGGAAT |
| RoRα-R | TGGTCCGATCAATCAAACAGTTC |
